# Depth dose perturbation by a hydrogel fiducial marker in a proton beam

**DOI:** 10.1120/jacmp.v16i1.5090

**Published:** 2015-01-08

**Authors:** Miao Zhang, Meral Reyhan, Leonard H. Kim

**Affiliations:** ^1^ Department of Radiation Oncology Rutgers Cancer Institute of New Jersey New Brunswick NJ USA

**Keywords:** fiducial marker, proton dose perturbation, hydrogel marker, gold marker

## Abstract

The purpose of this study was to evaluate proton depth dose perturbation caused by a radio‐opaque hydrogel fiducial marker. Electronic proton stopping powers in the hydrogel were calculated for energies 0.5–250 MeV, and Monte Carlo simulations were generated of hydrogel vs. gold markers placed at various water phantom depths in a generic proton beam. Across the studied energy range, the gel/water stopping power ratio was 1.0146 to 1.0160. In the Monte Carlo simulation, the hydrogel marker caused no discernible perturbation of the proton percent depth‐dose (PDD) curve. In contrast, the gold marker caused dose reductions of as much as 20% and dose shadowing regions as long as 6.5 cm. In contrast to gold markers, the radio‐opaque hydrogel marker causes negligible proton depth dose perturbation. This factor may be taken into consideration for image‐guided proton therapy at facilities with suitable imaging modalities.

PACS number: 87.55.Qr

## I. INTRODUCTION

Numerous studies have shown that fiducial markers can cause dose perturbations in a proton beam. A series of papers from M.D. Anderson have reported extensively on this topic for a variety of fiducial markers: gold, titanium, steel, carbon‐coated ${\text{ZrO}}_{2}$, tantalum, and others.^1^, ^2^, ^3^, ^4^, ^5^ Factors identified as influencing dose perturbation include marker composition, size, position, and orientation. In general, markers exhibiting the least dose perturbation are more susceptible to issues of imaging visibility and implantability due to their small size or composition. Commonly used gold markers are not recommended due to unacceptably large proton dose perturbations of 20%–80%, and the potential effect of fiducial markers on proton dosimetry should be a consideration in clinical practice.

**Figure 1:**
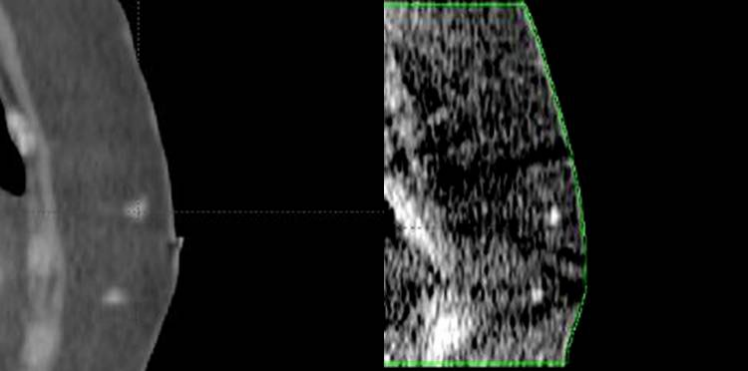
CT and kV CBCT images of two TraceIT markers in the breast. The CT image was acquired at 120 kVp using a GE LightSpeed with AEC; the CBCT image was acquired at 110 kVp, 259 mAs using a Varian OBI.

The TraceIT Tissue Marker (Augmenix Inc., Waltham, MA) is an iodinated polyethylene glycol (PEG) hydrogel fiducial marker. It has been shown to be clearly visible without artifact in 3D imaging modalities such as CT, MRI, CBCT, and ultrasound.^(6)^ It is injected by a physician using a syringe and 25‐gauge needle or cannula provided by the manufacturer to form a localized bleb in tissue. Representative CT and kV CBCT images acquired at our institution of 0.3 mL injections of the marker into a breast are shown in Fig. 1. In the CT image, the markers are approximately 275 HU. One notable feature of this marker is that it is temporary. The marker is absorbed over three months, so patients do not retain the marker after radiotherapy. In this study, the proton depth‐dose perturbation introduced by the TraceIT marker was investigated through stopping‐power calculation and Monte Carlo simulation.

## II. MATERIALS AND METHODS

Electronic (collisional) proton stopping power for the TraceIT gel was determined using the NIST computer program PSTAR for proton energies from 0.5 MeV to 250 MeV using an initial excitation energy for water of 75 eV. The composition of the TraceIT marker was specified as 90% water, 9.25% PEG, and 0.75% iodine by weight, with a density of 1.02 g/cm^3^ based on approximate information given by the manufacturer. Lacking information on PEG chain length, we assumed a value n=25, which well‐matches the proton stopping power for longer chain lengths and results in a small difference for smaller values. (Across the range of proton energies studied, the maximum stopping‐power difference between n=1 and n=25 was 0.001% or 0.04 MeV cm^2^/g.)

To illustrate proton depth‐dose perturbation introduced by the gel marker, the Geant4 Monte Carlo toolkit was used to simulate depth‐dose perturbations introduced by the TraceIT versus gold markers on a generic scattered proton beam in a $20\times 20\times 20\;{\text{cm}}^{3}$ water phantom. The beam was constructed by weight optimization of 27 individual proton beams to achieve a spread‐out Bragg peak (SOBP) dose plateau at depths between 8.2 cm and 18.7 cm in water. The mean energies of the individual proton beams ranged from 110 MeV to 170 MeV, with an interval of 2.5 MeV. We note that, though these initial energies are lower than those used to treat deep‐seated sites such as the prostate (∼200 MeV), the residual beam energy “seen” by markers in the uniform dose region will be comparable. The energy spread of each individual beam was modeled as a Gaussian with δ equal to 1% of the mean energy, comparable to a beam generated by a cyclotron with range shifter.^(7)^ Each individual beam was monodirectional, with a field size of $10\times 10\;{\text{cm}}^{2}$. The dose variation within the uniform dose region was 0.5%.

The simulated TraceIT marker was $1\times 1\times 1\;{\text{cm}}^{3}$. For simplicity, a cube shape was chosen, though in practice, the marker will not take this shape. The simulated gold marker was $0.1\times 0.1\times 0.3\;{\text{cm}}^{3}$ with a density of 19.3 g/cm^3^. The long axis of the gold marker was placed perpendicular to the direction of the beam, as this orientation has been shown to cause the least dose perturbation.^(5)^ Separate simulations were performed for markers placed at 10.0 cm and 16.5 cm depth. Finally, $2.8\times 10^{8}$ proton histories were simulated for each case, and all secondary particles were tracked.

## III. RESULTS

**Figure 2:**
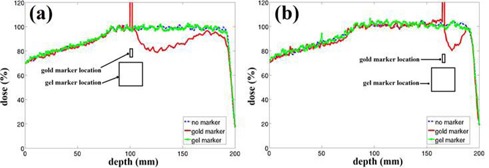
PDD of a simulated proton SOBP in a water phantom with no marker, gold marker, and TraceIT gel marker. placed at a depth of (a) 10.0 cm and (b) 16.5 cm.

Across the studied energy range, the gel/water electronic stopping‐power ratio was 1.0146 to 1.0160. Figure 2 shows simulated PDDs with and without each marker. A fine scoring resolution of $0.1\times 0.1\times 0.1\;{\text{cm}}^{3}$ was used to plot the curves with a statistical uncertainty of <2.5% (1 SD). The gold marker reduced proton dose by as much as 20% with a dose‐shadowing region as long as 6.5 cm with a >10% underdose. In contrast, no difference was observed between the PDDs with and without the hydrogel marker, even when the dose scoring resolution was decreased to $0.5\times 0.5\times 0.1\;{\text{cm}}^{3}$ to reduce statistical uncertainty to less than 0.5% (1 SD).

## IV. DISCUSSION & CONCLUSION

The TraceIT tissue marker has been evaluated favorably for imaging and implantation in a recent publication by de Souza Lawrence et al.^(6)^ One topic that was not reported on in that study was the expected lack of radiotherapy dose perturbation caused by the marker, which, as noted above, is composed >99% of water and PEG. Given the current literature on the effect of conventional fiducial markers on proton dosimetry and the search for better alternatives, proton radiotherapy seemed an appropriate scenario for study, though similar results would probably be obtained for many other radiotherapy modalities.

As expected, no discernible effect was observed, even from a large hydrogel marker on a proton PDD curve. This lack of perturbation may also allow greater confidence in analytical dose calculation algorithms used in treatment planning systems when hydrogel markers are used instead of metal markers, as such algorithms may underestimate heterogeneity effects from metal implants. The lack of dose perturbation in combination with reported artifact‐free imaging, ease of placement, and temporary lifespan^(6)^ suggests radiopaque hydrogel markers are candidates for use in image‐guided proton therapy as facilities adopt 3D imaging. However, because the markers are not easily visible in 2D radiographs,^(6)^ alternative solutions would be required for facilities using 2D imaging for image guidance or tracking. The paper by de Souza Lawrence et al.^(6)^ reports experience implanting hydrogel markers in thoracic sites such as the esophagus, mediastinum, and lung parenchyma, but published reports for other anatomical sites are currently lacking, though conference abstracts describe the use of hydrogel markers in the breast and bladder.^8^, ^9^ Further study of hydrogel markers in the clinic, including such issues as potential marker migration, effect on other treatment modalities, and optimal imaging techniques, would be desirable prior to widespread clinical use.

## ACKNOWLEDGMENTS

We thank Xiaohu Mo, Ph.D. of 21st Century Oncology for his help in generating the simulated proton beam, Patrick Campbell, Ph.D. of Augmenix, Inc. for technical information about the TraceIT tissue marker, and Stephen Seltzer, Ph.D. for his help generating proton stopping power using the PSTAR computer program.

## Supporting information

Supplementary Material
