# Supplementary material for: Depth dose perturbation by a hydrogel fiducial marker in a proton beam
Source: J Appl Clin Med Phys. 2015 Jan 8;16(1):373–6. doi: 10.1120/jacmp.v16i1.5090 (PMC5689967; doi:10.1120/jacmp.v16i1.5090)
Supplement: Supplementary file 1 — Supplementary Material [file ACM2-16-373-s001.docx]

Electronic Proton Stopping Power for TraceIT gel

| Energy | Electronic Proton Stopping Power | Ratio of Electronic Proton Stopping Power (TraceIT/Water) |
| --- | --- | --- |
| (MeV) | (MeV cm2/g) | (TraceIT MeV/cm/ Water MeV/cm) |
| 0.5 | 410.7 | 1.0148 |
| 0.55 | 386.75 | 1.0146 |
| 0.6 | 365.7 | 1.0147 |
| 0.65 | 347.07 | 1.0147 |
| 0.7 | 330.47 | 1.0147 |
| 0.75 | 315.59 | 1.0148 |
| 0.8 | 302.11 | 1.0147 |
| 0.85 | 289.95 | 1.0149 |
| 0.9 | 278.84 | 1.0147 |
| 0.95 | 268.64 | 1.0149 |
| 1 | 259.27 | 1.0148 |
| 1.25 | 221.66 | 1.0148 |
| 1.5 | 194.6 | 1.0153 |
| 1.75 | 173.98 | 1.0152 |
| 2 | 157.76 | 1.0152 |
| 2.25 | 144.59 | 1.0150 |
| 2.5 | 133.68 | 1.0153 |
| 2.75 | 124.47 | 1.0157 |
| 3 | 116.55 | 1.0152 |
| 3.5 | 103.66 | 1.0157 |
| 4 | 93.56 | 1.0154 |
| 4.5 | 85.421 | 1.0155 |
| 5 | 78.705 | 1.0154 |
| 5.5 | 73.06 | 1.0154 |
| 6 | 68.236 | 1.0155 |
| 6.5 | 64.061 | 1.0156 |
| 7 | 60.41 | 1.0155 |
| 7.5 | 57.188 | 1.0155 |
| 8 | 54.325 | 1.0156 |
| 8.5 | 51.76 | 1.0155 |
| 9 | 49.447 | 1.0156 |
| 9.5 | 47.351 | 1.0155 |
| 10 | 45.441 | 1.0156 |
| 12.5 | 37.967 | 1.0156 |
| 15 | 32.76 | 1.0157 |
| 17.5 | 28.912 | 1.0155 |
| 20 | 25.942 | 1.0158 |
| 22.5 | 23.576 | 1.0155 |
| 25 | 21.644 | 1.0155 |
| 27.5 | 20.034 | 1.0156 |
| 30 | 18.671 | 1.0157 |
| 35 | 16.486 | 1.0154 |
| 40 | 14.807 | 1.0157 |
| 45 | 13.475 | 1.0159 |
| 50 | 12.391 | 1.0160 |
| 55 | 11.491 | 1.0157 |
| 60 | 10.73 | 1.0153 |
| 65 | 10.079 | 1.0159 |
| 70 | 9.5155 | 1.0158 |
| 75 | 9.022 | 1.0158 |
| 80 | 8.5864 | 1.0158 |
| 85 | 8.1989 | 1.0158 |
| 90 | 7.8519 | 1.0158 |
| 95 | 7.5394 | 1.0159 |
| 100 | 7.2563 | 1.0158 |
| 105 | 6.9987 | 1.0159 |
| 110 | 6.7633 | 1.0158 |
| 115 | 6.5472 | 1.0158 |
| 120 | 6.3482 | 1.0159 |
| 125 | 6.1643 | 1.0158 |
| 130 | 5.9939 | 1.0159 |
| 135 | 5.8355 | 1.0159 |
| 140 | 5.6878 | 1.0159 |
| 145 | 5.5499 | 1.0158 |
| 150 | 5.4207 | 1.0158 |
| 155 | 5.2996 | 1.0159 |
| 160 | 5.1857 | 1.0158 |
| 165 | 5.0784 | 1.0159 |
| 170 | 4.9771 | 1.0159 |
| 175 | 4.8815 | 1.0159 |
| 180 | 4.7909 | 1.0159 |
| 185 | 4.7051 | 1.0159 |
| 190 | 4.6236 | 1.0160 |
| 195 | 4.5462 | 1.0158 |
| 200 | 4.4725 | 1.0158 |
| 205 | 4.4023 | 1.0159 |
| 210 | 4.3353 | 1.0159 |
| 215 | 4.2714 | 1.0158 |
| 220 | 4.2103 | 1.0160 |
| 225 | 4.1518 | 1.0158 |
| 230 | 4.0958 | 1.0160 |
| 235 | 4.0422 | 1.0158 |
| 240 | 3.9907 | 1.0159 |
| 245 | 3.9413 | 1.0160 |
| 250 | 3.8938 | 1.0158 |
